# Supplementary material for: Improved Detection of Molecular Markers of Atherosclerotic Plaques Using Sub-Millimeter PET Imaging
Source: Molecules. 2020 Apr 16;25(8):1838. doi: 10.3390/molecules25081838 (PMC7221983; doi:10.3390/molecules25081838)
Supplement: Supplementary file 1 [file molecules-25-01838-s001.pdf]

Supplementary data - **Improved detection of molecular markers of atherosclerotic plaques using sub-millimeter PET imaging**

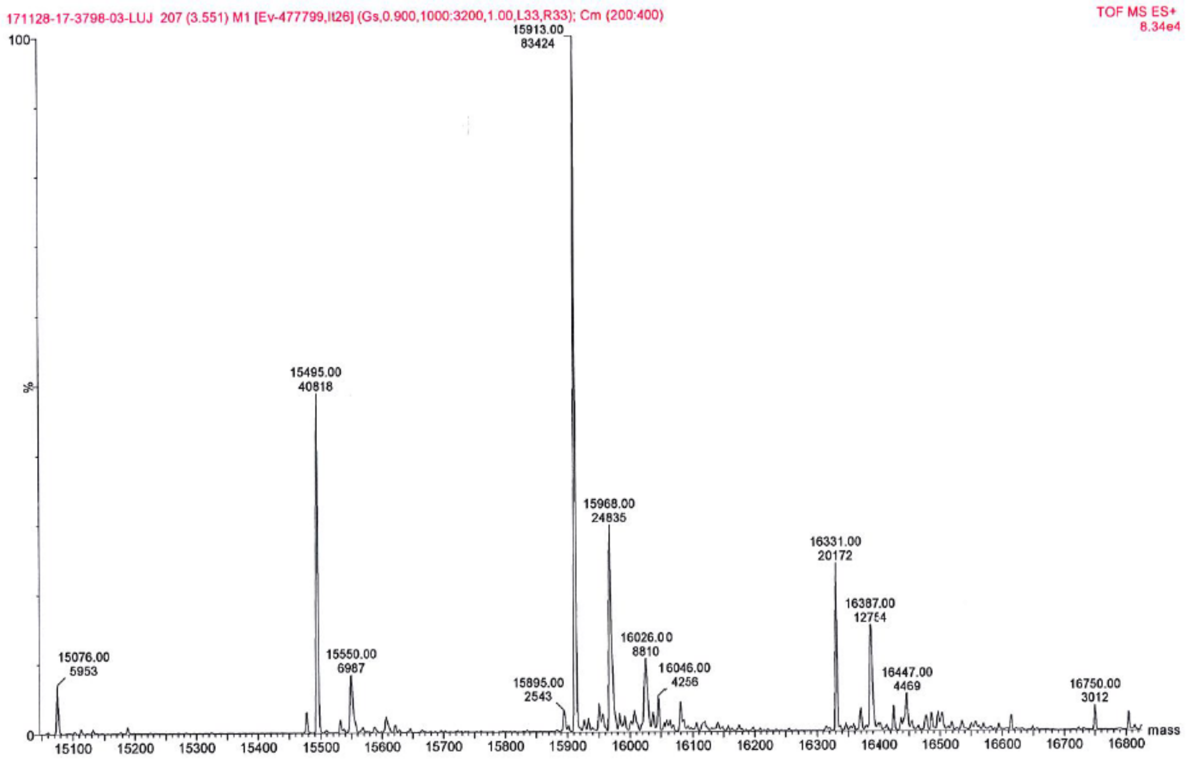

Figure 1: Mass determination analysis of the modified Nb-(RESCA)$_n$ showing major peaks of 15495 ± 2 Da (n = 2, calculated = 15496 Da) and 15913 ± 2 Da (n = 3, calculated = Mw = 15076 Da, measured = 15076±2 Da). Minor peaks were measured: 15076 ± 2 Da (n = 1, calculated = 15915 Da), 16331 ± 2 Da (n = 4, calculated = 16334 Da), 16750 ± 2 Da (n = 5, calculated 16754 Da).

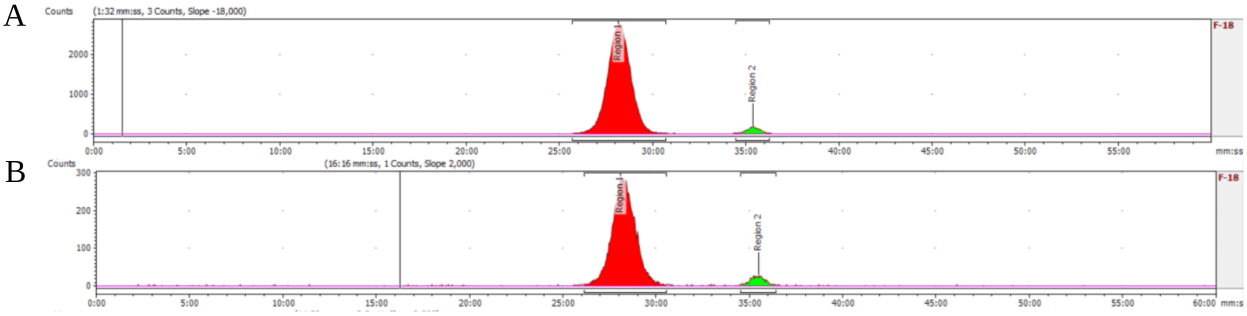

Figure 2: SEC profile of [^{18}F]AlF(RESCA)-cAbVCAM1-5 Nb (A) after 1h20 in human serum at 37°C. [^{18}F]AlF(RESCA)-cAbVCAM1-5 Rt = 28.0 min (96%), free [^{18}F]F$^-$ or

[^{18}F]AlF Rf = 35.2 min (4%). (A) after 2h30 in human serum at 37°C. [^{18}F]AlF(RESCA)-cAbVCAM1-5 Rt = 28.0 min (94%), free [^{18}F]F$^-$ or [^{18}F]AlF Rf = 35.3 min (6%).

	**[^{18}F]AlF-RESCA-cAbVCAM1-5**		**[^{18}F]AlF-RESCA-cAbVCAM1-5 + Blocking**	
	Average	STDEV	Average	STDEV
Blood	0,18	0,06	0,17	0,09
Liver	0,18	0,03	0,17	0,05
Spleen	1,01	0,34	0,34	0,14
Pancreas	0,06	0,02	0,07	0,04
Kidney R	13,68	3,44	21,09	9,83
Kidney L	14,32	4,05	21,77	11,67
Stomach	0,17	0,05	0,16	0,11
Small Intestine	0,65	0,27	0,71	0,61
Large Intestine	0,63	0,57	0,24	0,38
Heart	0,10	0,02	0,09	0,03
Lungs	0,42	0,11	0,29	0,19
Thymus	0,32	0,09	0,22	0,06
Muscle	0,22	0,30	0,09	0,05
Bone	1,13	0,33	0,96	0,33
Lymph Node	0,54	0,15	0,33	0,22
Fat	0,08	0,07	0,06	0,02

Table 1: Biodistribution. Average of %IA/g for each organ or tissue, with the standard deviation (STDEV) for both groups (N = 6 / group) injected with [^{18}F]AlF-RESCA-cAbVCAM1-5 or [^{18}F]AlF-RESCA-cAbVCAM1-5 + Blocking (control).
